# Surgical Resolution of Patients With Hypertrophy of Papillary Muscles in Hypertrophic Cardiomyopathy

**DOI:** 10.7759/cureus.66827

**Published:** 2024-08-13

**Authors:** Rutilio Daniel Jiménez Espinosa, Jesús Saucedo Castillo, Luis León Hernández Trejo, Dalia Chacón Martell, Denise Guzmán Lázaro, Ana Luisa Hernández Pérez

**Affiliations:** 1 Department of Cardiothoracic Surgery, Hospital de Cardiología, Centro Médico Nacional Siglo XXI, Instituto Mexicano del Seguro Social, Mexico City, MEX; 2 Department of Anesthesiology, Hospital de Cardiología, Centro Médico Nacional Siglo XXI, Instituto Mexicano del Seguro Social, Mexico City, MEX; 3 Department of Anesthesiology, Centro Médico ABC, Mexico City, MEX

**Keywords:** morrow procedure, septal myectomy, papillary muscle hypertrophy, septal hypertrophy, hypertrophic cardiomyopathy

## Abstract

Hypertrophic cardiomyopathy encompasses a broad spectrum of muscular diseases that involve not only the interventricular septum and the left ventricular outflow tract but also the papillary muscles and the mitral valve apparatus. This article presents the successful surgical treatment of two patients with generalized hypertrophic cardiomyopathy with hypertrophy of the papillary muscles without severe septal hypertrophy: one with a history of unsuccessful chemical ablation and coronary disease following an interventional event, and another young patient without a history of chronic degenerative diseases who developed hypertrophic cardiomyopathy during her third pregnancy. Both patients with left ventricular outflow tract gradients greater than 55 mmHg and those who underwent surgical treatment had a gradient of less than 10 mmHg.

## Introduction

Hypertrophic cardiomyopathy (HCM) is defined as “a disorder in which the heart muscle is structurally and functionally abnormal, in the absence of coronary artery disease, hypertension, valvular disease, and congenital heart disease sufficient to cause the observed myocardial abnormality.” In other words, only the muscle is affected (hypertrophic) and creates a gradient, unlike aortic stenosis, where valve obstruction causes ventricular hypertrophy. In hypertrophic cardiomyopathy, the muscle becomes thickened without valvular disease, as in systemic arterial hypertension, where increased vascular resistance leads to ventricular hypertrophy [1].

HCM is a common genetic heart disease reported worldwide. It has an autosomal dominant pattern and is equally distributed by sex, although women are diagnosed less frequently than men [2]. Variants can include basal, midventricular, apical, or diffuse types. It is associated with a variety of clinical presentations ranging from asymptomatic to sudden death [3]. The most common morphology consists of hypertrophy of the basal interventricular septum and concomitant systolic anterior motion (SAM) of the anterior leaflet of the mitral valve, resulting in obstruction of the left ventricular outflow tract (LVOT) [4]. From a surgical perspective, patients with classic HCM can be subclassified based on the site of maximum septal hypertrophy, although other segments of the wall are generally hypertrophied as well [1,5].

The surgical treatment of patients with obstructive HCM can be extremely challenging. Resolution of LVOT obstruction in these patients is often achieved by performing septal myectomy. However, in many cases, septal reduction alone is not sufficient to relieve the obstruction. Interventions involving the subvalvular apparatus, including abnormal chordae tendineae and anomalous papillary muscles, are often needed [6]. In such cases, we decide to perform a Morrow-type procedure (septal myectomy) combined with papillary muscle resection and mitral valve replacement.

## Case presentation

Patient 1

**Figure 1:**
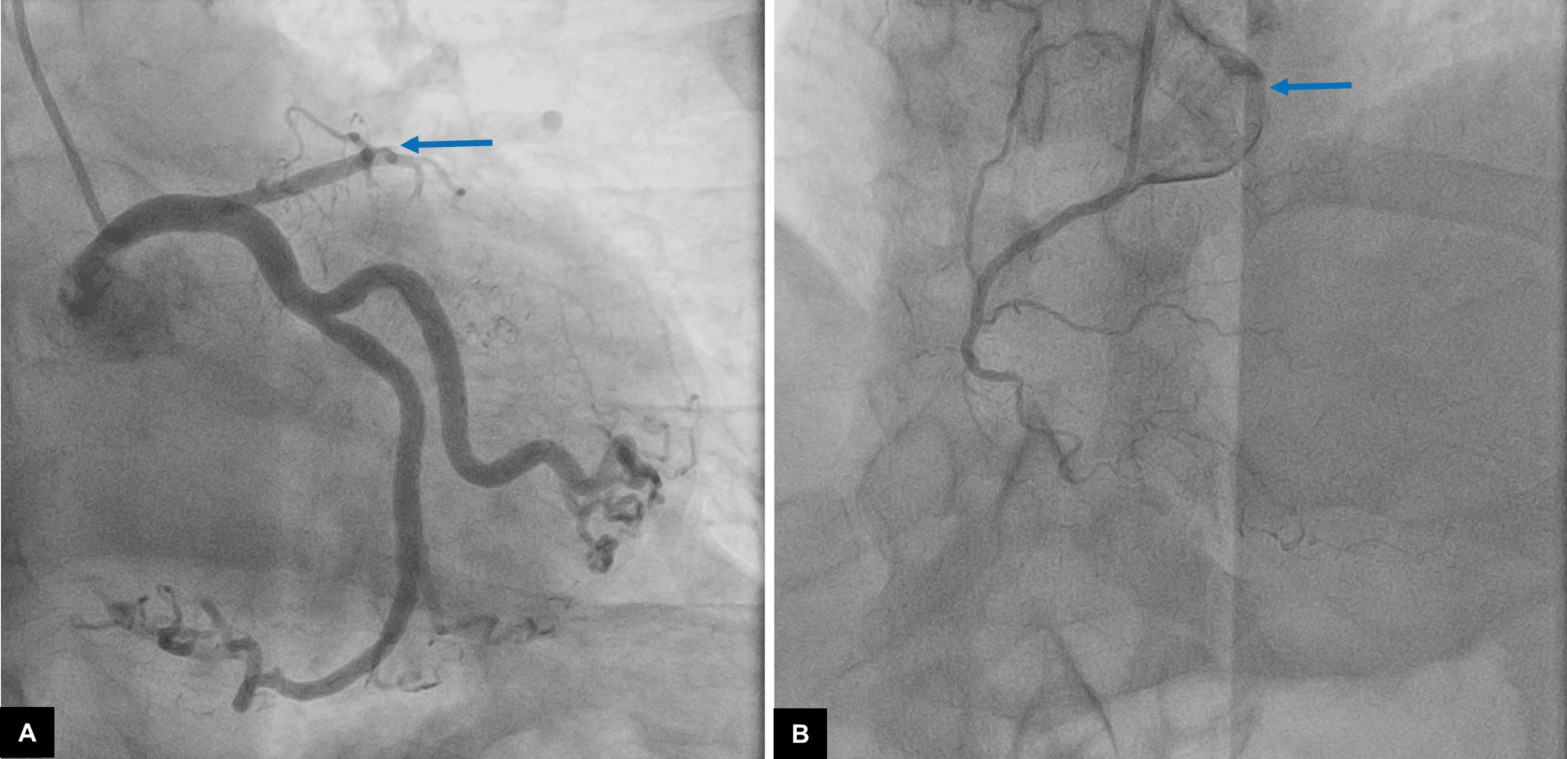
Cardiac catheterization. (A) Chronic occlusion of the left anterior descending artery secondary to chemical ablation (blue arrow). (B) The right coronary artery with reduced flow consistent with chronic occlusion in the proximal third (blue arrow).

A 63-year-old male with a history of HCM underwent alcohol ablation in 2002. The gradient before alcohol ablation was 40 mmHg which did not improve after the procedure and had increased to 55 mmHg by the time of the surgical decision. In December of the same year, the patient underwent cardiac catheterization and was diagnosed with chronic coronary syndrome, with the presence of multivessel arterial disease mainly in the left anterior descending artery with complete chronic occlusion in its proximal segment, as well as in the right coronary artery with complete chronic occlusion in its proximal segment (Figures 1A, 1B).

**Figure 2:**
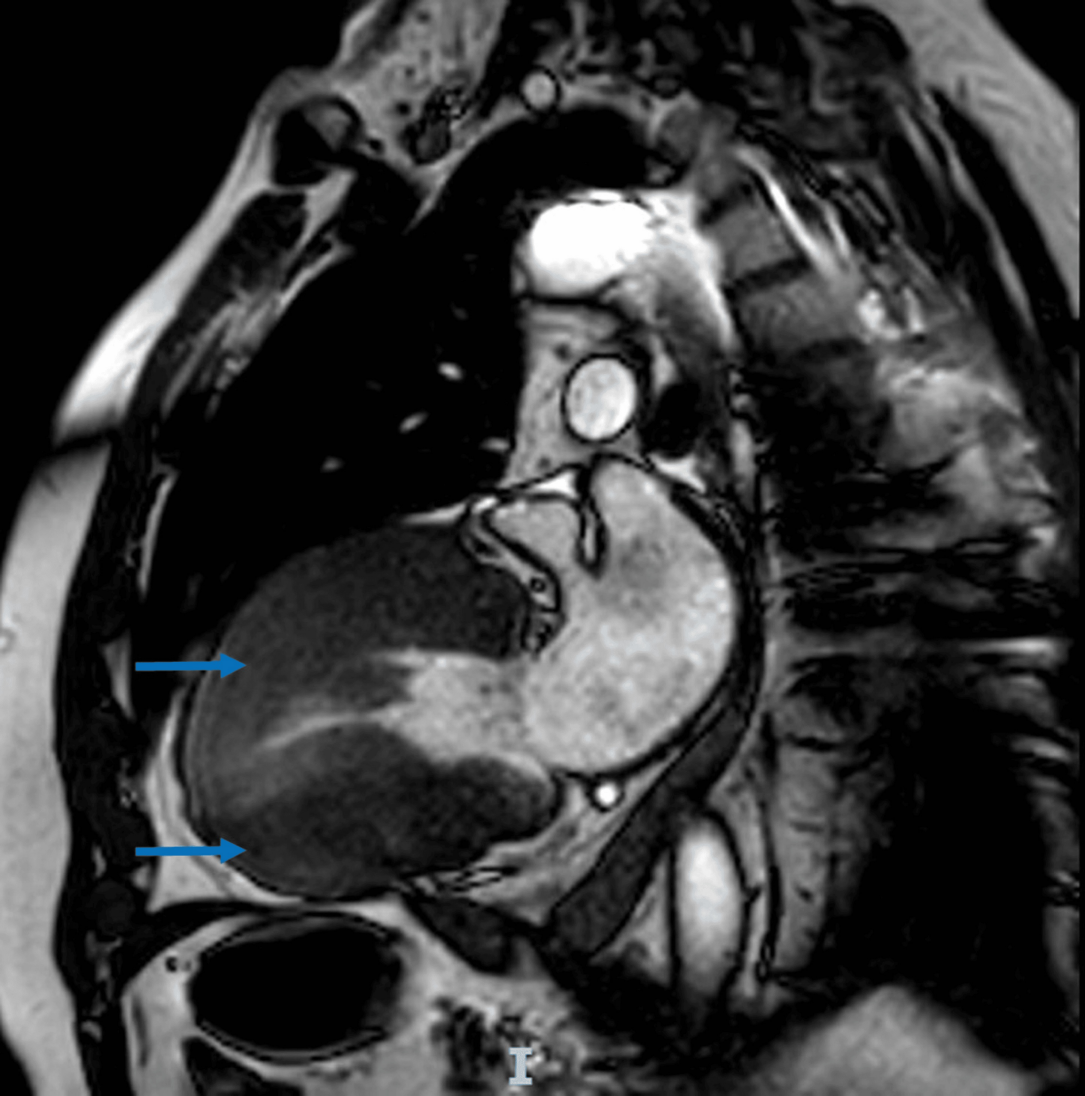
Hypertrophic cardiomyopathy of the left ventricle, anterior and posterior walls (blue arrows).

There was coronary ectasia of the circumflex artery in addition to the previously known diagnosis of asymmetric septal myocardiopathy with a 55 mmHg LVOT gradient. While at rest, he experienced angina-like pain and ST-segment depression in V5-V6 on the electrocardiogram (ECG). He was hospitalized and treated pharmacologically, and the symptoms improved. During this hospital stay, a 99mTc-sestamibi single-photon emission computed tomography/computed tomography cardiac scan was performed, which revealed moderate inferolateral ischemia, apical and septal infarction with mild residual ischemia, a left ventricular ejection fraction (LVEF) of 51% during stress and 59% at rest, septal thickening of 17 mm with evidence of LVOT obstruction and papillary muscle thickening of 20 mm, both with moderate MR. Cardiac magnetic resonance imaging (MRI) revealed a septal thickness of 22 mm and posterior papillary muscle thickening of 20 mm (Figure 2). The patient was considered for the Morrow procedure, mitral valve apparatus resection, and mitral valve replacement. For coronary artery disease, medical management was decided based on antiplatelet therapy and statins.

The surgical procedure was performed through a complete median sternotomy. The patient underwent total cardiopulmonary bypass with bicaval venous drainage. Arterial cannulation was performed at the aortic root, maintaining a temperature of 32 degrees. An incision was made for the high transeptal atrial approach to visualize the mitral valve and left ventricular cavity. Both thickened papillary muscles were identified and resected. Mitral valve replacement was performed with a Saint Jude 31 mechanical prosthesis, followed by aortotomy for myectomy starting from below the commissure between the right and left leaflets toward the apex. Aortorrhaphy and atrial closure were performed. The patient was successfully weaned off bypass with minimal inotropic support.

The maximum left ventricular outflow tract gradient was 9 mmHg one month after the surgical procedure. At the one-year follow-up, the patient still had adequate functional status (New York Heart Association (NYHA) l/ll).

Patient 2

**Figure 3:**
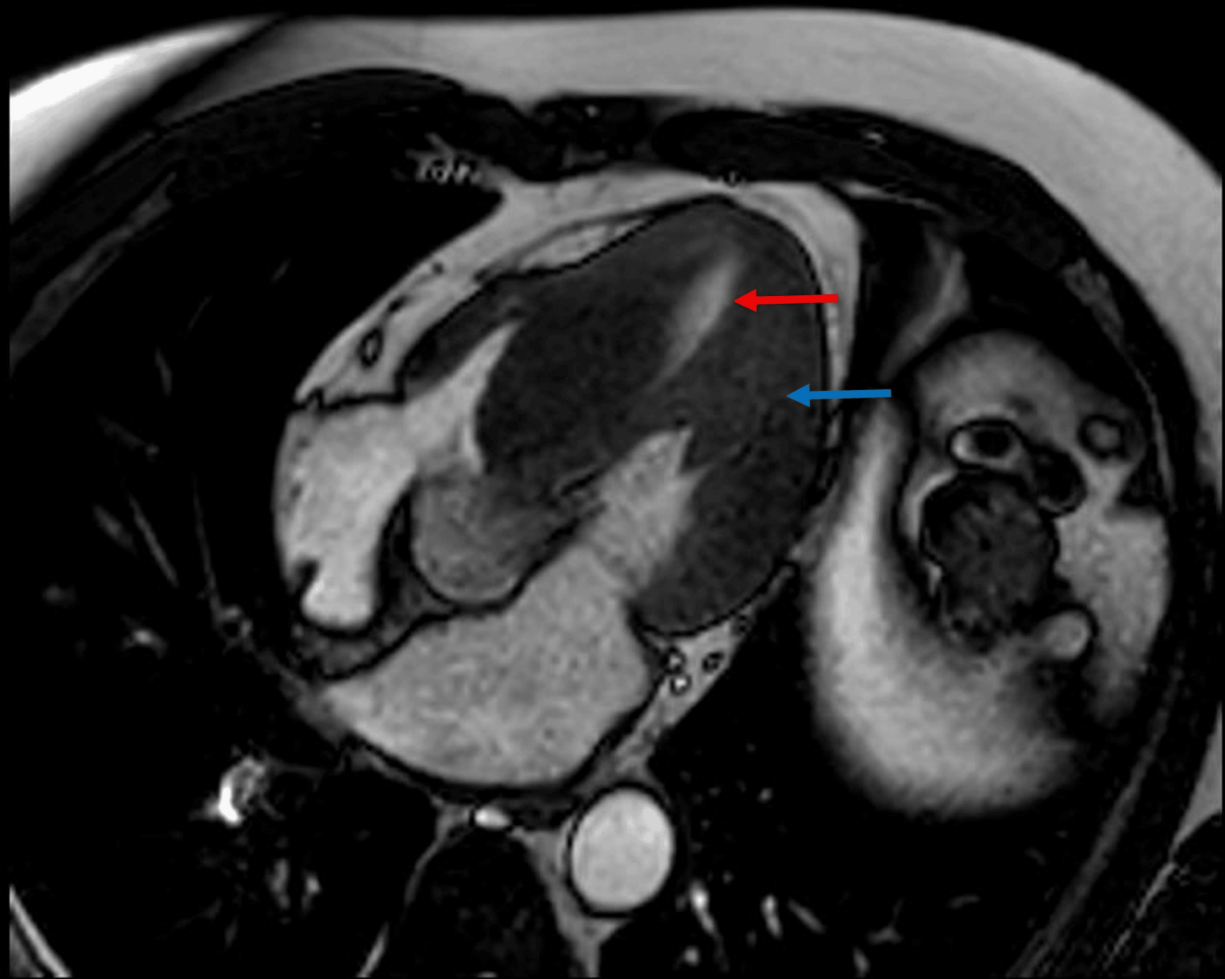
Magnetic resonance imaging showing a residual ventricular cavity (red arrow) secondary to the thickening of papillary muscles and the septum (blue arrow).

A 33-year-old female patient had no history of chronic degenerative diseases, except for a cesarean section during her third pregnancy due to preeclampsia, without subsequent antihypertensive treatment. Her symptoms began in July 2023, with exertional dyspnea and syncope, along with dyspnea at rest. An echocardiogram revealed asymmetric septal hypertrophy with an obstructive gradient of 103 mmHg at the LVOT due to SAM of the mitral valve, an LVEF of 63%, and severe mitral regurgitation. Coronary angiography revealed no lesions. MRI revealed a septal thickness of 25 mm and posterior papillary muscle thickening of 20 mm (Figure 3).

**Figure 4:**
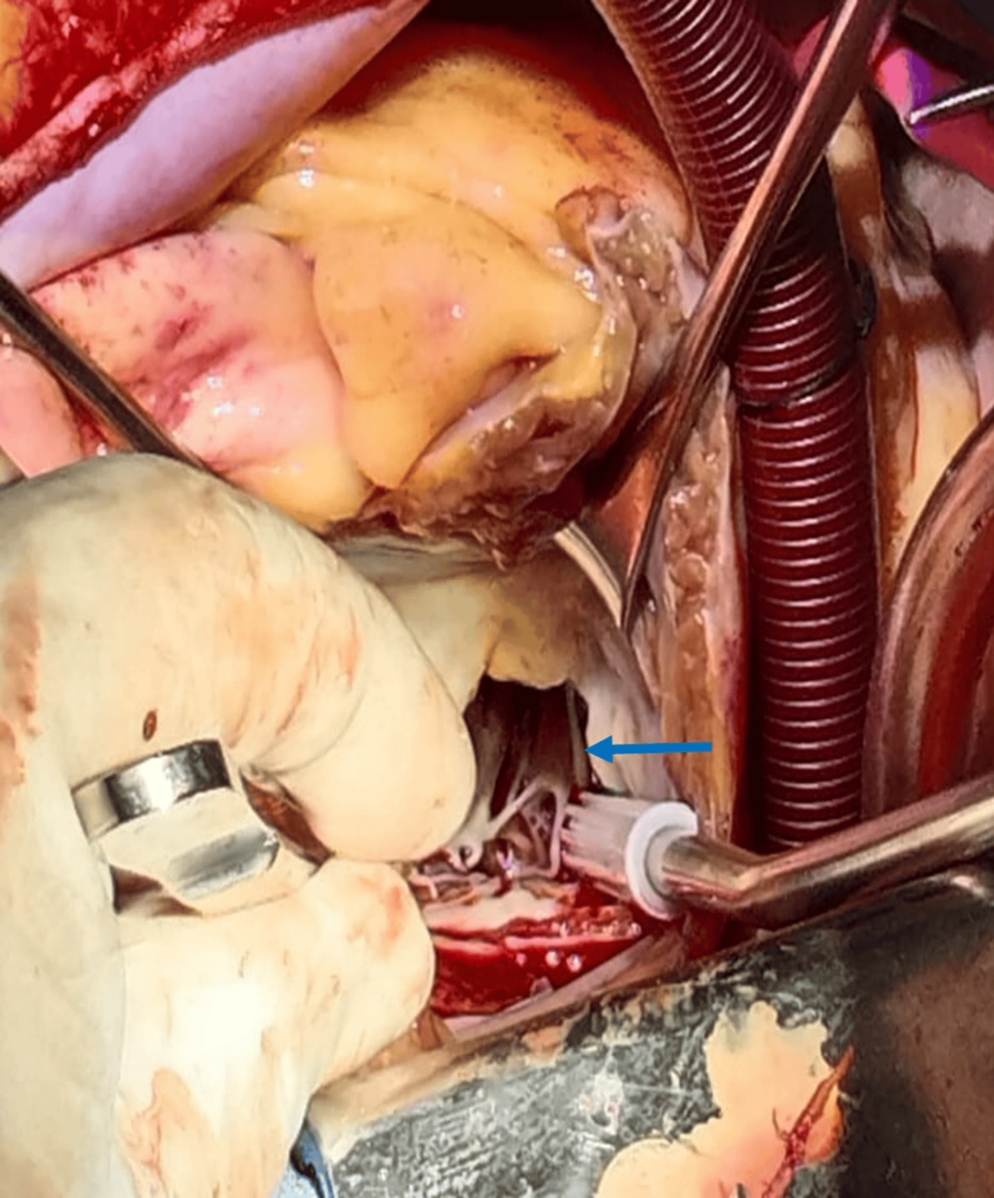
Surgical view of the thickened posterior papillary muscle (blue arrow).

**Figure 5:**
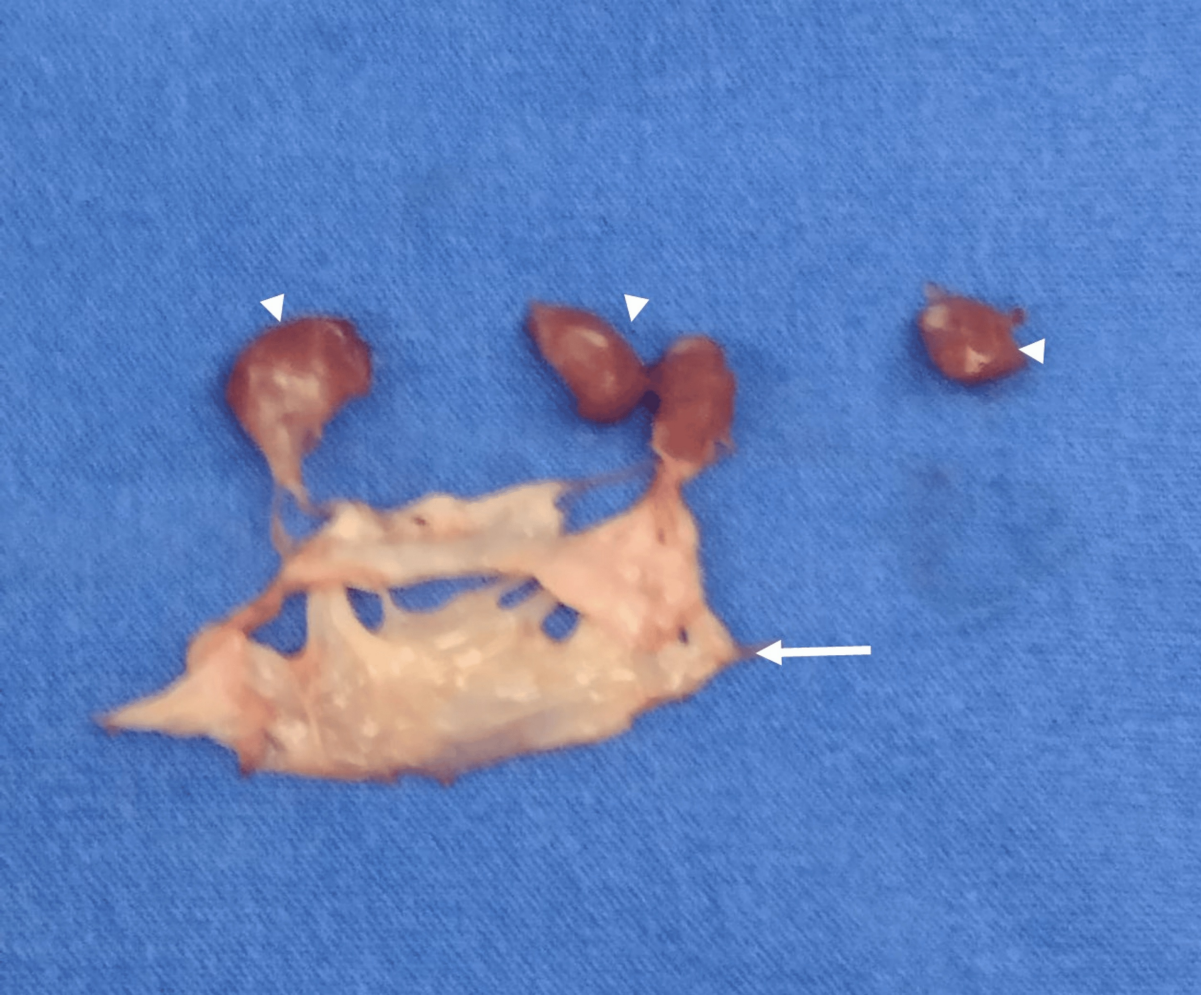
Resection of the mitral valve (white arrow) with subvalvular apparatus, including resection of both papillary muscles, each measuring 17 mm (arrowheads).

She underwent the Morrow procedure and mitral valve apparatus resection with mitral valve prosthesis implantation via the same surgical approach as in the previous case. Mitral valve replacement was performed with a Saint Jude 29 mechanical prosthesis. The only difference was the bifid and thickened posteromedial muscle necessitating resection (Figures 4, 5).

The postoperative maximum LVOT gradient was 6 mmHg one month after surgery. After six months of follow-up, a new echocardiogram was performed, which revealed the proper function of the valve prosthesis in the mitral position without obstructive gradients of the LVOT. Regarding his functional status, he remains in the NYHA II functional class.

## Discussion

The anatomical characteristics of different types of HCM are presented in Table 1 [1,5].

**Table 1:** Anatomical characteristics of different types of hypertrophic cardiomyopathy. LVOT: left ventricular outflow tract; SAM: systolic anterior motion; LV: left ventricle

Type	Characteristics	Result
Subaortic	Hypertrophy of the basal interventricular septum	Obstruction of LVOT by SAM
Midventricular	Midseptal hypertrophy	Formation of a crescent-shaped cavity in the LV may be associated with SAM and obstruction to the LVOT
Apical	Predominantly apical distribution of hypertrophy	Reduced size of the apical region of the left ventricular cavity

HCM is defined as an increase in the thickness of the left ventricle wall, with or without right ventricular hypertrophy, or a mass that is not solely explained by abnormal loading conditions or, specifically, in an adult [7]. HCM is defined as a left ventricle wall thickness ≥15 mm in any myocardial segment that cannot be solely explained by loading conditions. Minor degrees of myocardial wall thickening, between 13 and 14 mm, necessitate evaluation of other features, including family history, genetic findings, and ECG anomalies. In children, the diagnosis of HCM requires a left ventricle wall thickness exceeding two standard deviations above the expected mean (Z score >2) [8].

The approach to HCM is based on the interpretation of clinical findings and imaging studies to suspect and ultimately establish an etiological diagnosis based on the phenotype to guide specific disease treatment [9].

Noninvasive imaging modalities, including ultrasound-based techniques, cardiac MRI, CT, and nuclear techniques such as positron emission tomography and scintigraphy, are crucial for the diagnosis and monitoring of patients with cardiomyopathy [10].

Some individuals with subtle structural anomalies associated with cardiomyopathy remain asymptomatic and have a normal life expectancy; however, others may develop symptoms, often many years after the disease manifests or is identified through ECG or imaging studies. The main abnormality in systole is the obstruction of left ventricular outflow caused by hypertrophy of the superior interventricular septum, narrowing the outflow tract and setting the stage for Venturi forces, which causes anterior systolic motion of the mitral valve leaflets. This was evident in these patients.

It is essential to include cardiac scintigraphy in imaging studies because it allows the identification of the most significant thickness of the septum, which is not always definitive in echocardiography.

The timing and duration of contact between the mitral valve and interventricular septum determine the magnitude of the pressure gradient. This gradient is defined as an instantaneous maximum Doppler gradient in the LVOT of ≥30 mmHg, but the threshold for invasive treatment is typically considered to be ≥50 mmHg. Most patients with a resting or provoked maximum gradient in the LVOT of <50 mmHg should be managed according to the recommendations for nonobstructive HCM. However, in a very small number of selected cases with gradients in the LVOT between 30 and 50 mmHg and without any other apparent cause of symptoms, invasive gradient reduction may be considered. Mitral regurgitation invariably accompanies LVOT obstruction [11].

Invasive treatment should be considered to reduce LVOT obstruction in patients with a gradient ≥50 mmHg, severe symptoms considered by the NYHA as functional classes III-IV, and/or recurrent unexplained syncope or effort-related syncope despite maximally tolerated pharmacological treatment. This was the case for the male patient with a 55 mmHg gradient and the female patient with a 103 mmHg gradient.

Invasive therapy may also be considered in patients with mild symptoms (NYHA class II) refractory to medical treatment who have a resting or provoked maximum gradient of ≥50 mmHg (exercise or Valsalva) and moderate-to-severe mitral regurgitation related to systolic anterior motion. Atrial fibrillation (AF) or moderate-to-severe left atrial dilation can also be considered in expert centers with low demonstrated procedure complication rates [12].

According to the international literature, surgical treatment eliminates or substantially reduces LVOT gradients in more than 90% of cases, reduces systolic mitral regurgitation related to anterior motion, and improves exercise capacity and symptoms. A long-term symptomatic benefit is achieved in >80% of patients whose long-term survival is comparable to that of the general population. Preoperative determinants of a good long-term outcome include age <50 years, left atrial size <46 mm, absence of AF, and male sex.

In patients with intrinsic/primary mitral valve disease or marked elongation of the mitral leaflet and/or moderate-to-severe mitral regurgitation, septal myectomy can be combined with mitral valve repair or replacement. The most common postsurgical complications are as follows:

1. Ventricular septal defect: In this procedure, there is a possibility of creating a ventricular septal defect after septal myectomy, although the occurrence rate also depends on the medical center’s experience with the procedure. With current extended septal myectomy methods, the risk of ventricular septal defects is low (<1%) in specialized centers, even those with thin septa (<18 mm). If this consequence occurs, addressing the ventricular septal defect through right ventriculotomy via patch repair is suggested [3].

2. Aortic valve injury: Transaortic septal myectomy can lead to aortic valve injury, which is a significant lesion, although it is often overlooked.

The international literature reports an incidence rate between 2% and 36%. While aortic regurgitation may initially be trivial and well tolerated, some patients may require later repair. In a survey reported by the American College of Surgeons, an incidence rate of 1.5% of unplanned aortic valve replacement during septal myectomy was noted [3].

In these two cases, with moderate and severe mitral regurgitation along with papillary muscle thickening, resection of the entire mitral valve apparatus was performed instead of repair. This decision was made because the septum and posterior wall are not as thick as the papillary muscles, posing a risk of a significant residual LVOT gradient.

A key technical point in surgical resection is extending the apical septal excision beyond the anticipated hypertrophic surface, ensuring sufficient removal of hypertrophic tissue toward the apex of the left ventricle. Inadequate resection may lead to residual obstruction and gradient postoperatively, which is the most common reason for inadequate septal myectomy and a residual gradient.

This approach was successful, as the postoperative maximum LVOT gradient was 6 mmHg for the female patient and 9 mmHg for the male patient, indicating clinical improvement. Both patients remain asymptomatic from a cardiovascular perspective, whereas in another scenario, a sudden death event could have been expected.

## Conclusions

HCM is a genetic cardiac disease characterized by structural and functional abnormalities of the heart muscle. The surgical indication is determined by an LVOT gradient greater than 50 mmHg accompanied by severe symptoms. In certain patients, a surgical procedure involving septal myectomy, also known as the Morrow procedure, may not be sufficient. Therefore, we perform a procedure that, in addition to septal myectomy, combines the resection of the papillary muscle and valve mitral replacement, which can offer better results in reducing the LVOT gradient and consequently alleviating symptoms, as observed in the cases presented.
